# Cannabis Use during Adolescent Development: Susceptibility to Psychiatric Illness

**DOI:** 10.3389/fpsyt.2013.00129

**Published:** 2013-10-14

**Authors:** Benjamin Chadwick, Michael L. Miller, Yasmin L. Hurd

**Affiliations:** ^1^Fishberg Department of Neuroscience, Friedman Brain Institute, Icahn School of Medicine at Mount Sinai, New York, NY, USA; ^2^Department of Psychiatry, Icahn School of Medicine at Mount Sinai, New York, NY, USA; ^3^James J. Peters VA Medical Center, Bronx, NY, USA

**Keywords:** cannabis, drug addiction, negative affect, schizophrenia, adolescent

## Abstract

Cannabis use is increasingly pervasive among adolescents today, even more common than cigarette smoking. The evolving policy surrounding the legalization of cannabis reaffirms the need to understand the relationship between cannabis exposure early in life and psychiatric illnesses. cannabis contains psychoactive components, notably Δ^9^-tetrahydrocannabinol (THC), that interfere with the brain’s endogenous endocannabinoid system, which is critically involved in both pre- and post-natal neurodevelopment. Consequently, THC and related compounds could potentially usurp normal adolescent neurodevelopment, shifting the brain’s developmental trajectory toward a disease-vulnerable state, predisposing early cannabis users to motivational, affective, and psychotic disorders. Numerous human studies, including prospective longitudinal studies, demonstrate that early cannabis use is associated with major depressive disorder and drug addiction. A strong association between schizophrenia and cannabis use is also apparent, especially when considering genetic factors that interact with this environmental exposure. These human studies set a foundation for carefully controlled animal studies which demonstrate similar patterns following early cannabinoid exposure. Given the vulnerable nature of adolescent neurodevelopment and the persistent changes that follow early cannabis exposure, the experimental findings outlined should be carefully considered by policymakers. In order to fully address the growing issues of psychiatric illnesses and to ensure a healthy future, measures should be taken to reduce cannabis use among teens.

## Introduction

**Figure 1:**
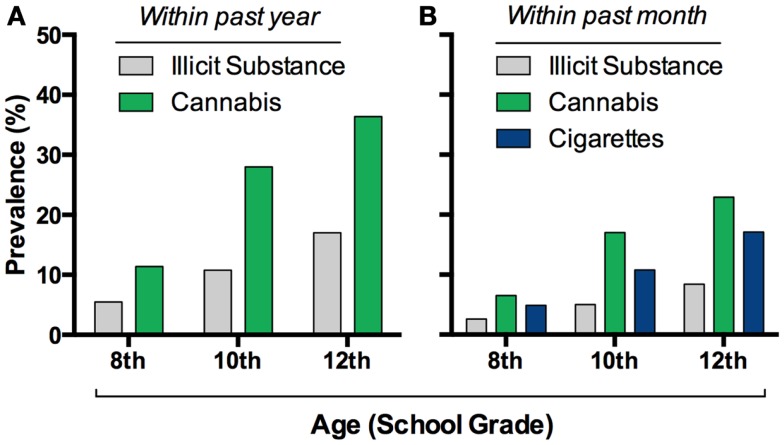
**Cannabis consumption is widespread in adolescents**. Prevalence of this drug’s intake exceeds other illicit drug’s in eighth through twelfth graders in the USA **(A)**, and it recently surpassed cigarette use **(B)**. Graphs based on data adapted from Johnston et al. (1)**(A,B)**.

**Figure 2:**
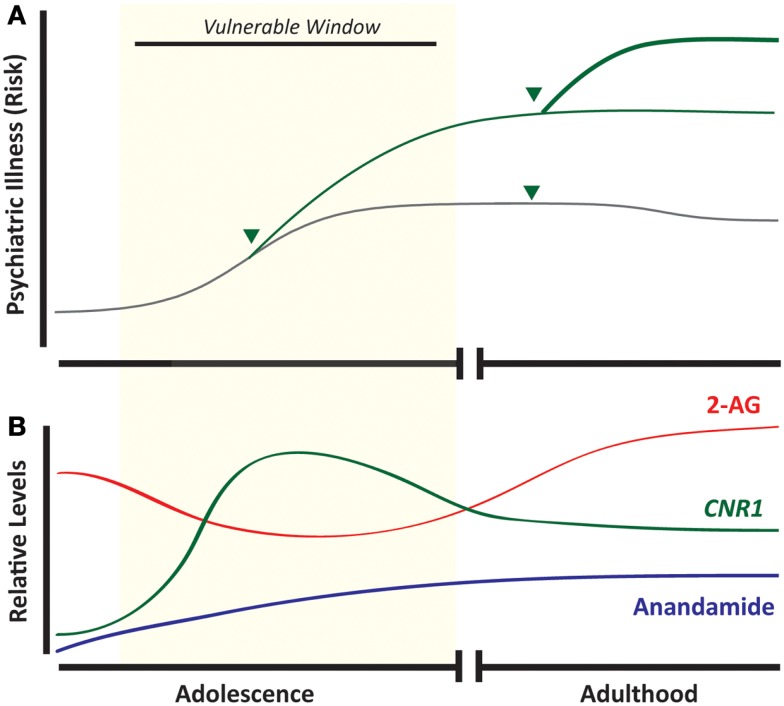
**Developmental cannabis increases vulnerability to psychiatric disease and overlaps with ontogenic changes in the endocannabinoid system**. Adolescence is associated with an increased incidence of psychiatric illness, and exposure to cannabis (arrow head) during this developmental window strongly predicts subsequent development of mood disorders, addictive disorders, and schizophrenia **(A)**. Components of the endocannabinoid system appear as early as embryonic life, but maximal *CNR1* mRNA expression occurs during adolescence **(B)**. (Green line = cannabis-exposed and gray line = unexposed individuals.)

*Cannabis sativa* is grown worldwide for its production of Δ^9^-tetrahydrocannabinol (THC), a psychoactive compound found in the recreational drugs marijuana and hashish. The pervasiveness of this drug worldwide, along with its relatively low lethality, has led many to believe that it is of little harm. Indeed, the use of cannabis currently exceeds that of tobacco smoking among adolescents in the United States (1) (Figure 1). Whether cannabis is harmless, and without significant physiological or mental health impact, is actively debated. Unfortunately, these discussions are often not guided by evidence-based data. Research focused on the relationship between cannabis and mental health is thus important especially considering that psychiatric illnesses are complex disorders with multiple factors contributing to vulnerability and eventual expression of the illness. Based on the accruing data to date outlined in this review, developmental cannabis exposure is an important contributing factor to psychiatric vulnerability (Figure 2A).

## Cannabis and Developmental Pattern of Use

Psychiatric illnesses are developmental in nature – the 12-month prevalence of any psychiatric illness is ∼40% in adolescents (2), but ∼25% in adults (3) – making it significantly germane to the strong developmental pattern of cannabis use. A plethora of studies and national surveys monitored the patterns of cannabis use in multiple ethnic and geographic populations worldwide. In the United States, cannabis use is highly prevalent during adolescence (Figure 1), the developmental period when most people initiate use. There are over 6000 first-time cannabis users per day in the US, over 60% of which are under the age of 18 (4). Approximately 34–45% of ninth through twelfth graders reported cannabis use at least once in their lifetime and the pattern of subsequent use appears more or less intermittent with 23% of 12 graders reporting use in the past month (1, 5, 6). Data from wave I–III of the National Longitudinal Study for Adolescent Health recapitulate this pattern of wide spread yet occasional use in adolescents. While the majority of teens have infrequent use, still a significant percentage, 6.6%, report daily use. Determining the long-term impact of occasional and heavy cannabis use during active periods of brain development, such as adolescence, is of critical importance. To provide such insights, data garnered from epidemiological and experimental studies is reviewed in this article. The emerging evidence strongly suggests that cannabis exposure during adolescence increases an adult’s individual vulnerability to drug addiction and schizophrenia and may also produce long-lasting effects on anxiety and mood disorders.

## Endocannabinoid System

The psychoactive effects of cannabis, principally mediated by THC, occur via its interaction with the endocannabinoid system, which regulates numerous biological processes involved in development and neuroplasticity. The endocannabinoid system consists of lipid-derived ligands, receptors, and enzymes that orchestrate intercellular communication and intracellular metabolism. The most characterized endocannabinoid ligands – or endocannabinoids (eCBs) – include 2-AG and anandamide, which are presumably synthesized via phospholipase-mediated pathways. At least two G-protein coupled receptors, referred to as cannabinoid receptor-1 (CB_1_R) and -2 (CB_2_R), interact with these ligands. Additionally, recent evidence suggests that eCBs bind to ligand-gated channels, particularly TRPV1. In regard to the ligands, eCBs are synthesized from membranous precursors and immediately diffuse to nearby cannabinoid receptors, classically expressed on pre-synaptic terminals. Following these events, co-expressed enzymes, such as monoacylglycerol lipase (MGLL), α-β-hydrolase domain 6 (ABHD6), and fatty acid amide hydrolase (FAAH), degrade the ligand to terminate its signal (7, 8). Tightly regulated biosynthetic and degradative pathways ensure proper signaling throughout development, and the correct function of these processes depends on the temporal and spatial patterning of this system. Exogenously consumed cannabis produces supraphysiological effects at eCB-targeted receptors and thus usurp the normal endocannabinoid system (9).

The endocannabinoid system is critical for neurodevelopment and as such is present in early development, and maintains expression throughout life (Figure 2B), exhibiting a broad spatial distribution to regulate synaptic plasticity (10, 11). The CB_1_R is found in numerous central nervous system structures as early as the eleventh embryonic day, and throughout the embryonic period this receptor is expressed in subcortical and cortical regions (12). In cortical projection neurons, CB_1_R and local eCBs facilitate the fasciculation of descending efferents and thalamic afferents, orchestrating the tight coupling of these two tracts (13). During adolescence, the endocannabinoid system still facilitates neurodevelopment through its intricate involvement in neuroplasticity and synaptic function. Receptor levels of CB_1_R in the prefrontal cortex and striatum fluctuate during adolescence depending on the specific brain region. For instance, there is a rapid, sustained increase in cannabinoid receptor binding during adolescence, particularly in the striatum, that is substantially reduced (by half) in early adulthood (14). In addition, the expression of the CB_1_R gene (*Cnr1*) is highest during adolescence and gradually decreases by adulthood with the greatest decreases observed in limbic-related cortical regions such as the cingulate, prelimbic, and infralimbic cortices (15). Concomitant to developmental changes in the CB_1_R, levels of anandamide and 2-AG, as well as FAAH enzymatic activity, fluctuate throughout adolescence in a region- and time-specific manner (16, 17). The distinct changes in CB_1_R and other components of the eCB system during adolescence, some of which occur during a narrow time window, suggest that certain phases during this dynamic ontogenic period may incur different sensitivity to cannabis exposure. These observations highlight the fact that despite significant studies of CB_1_R in the adult brain, there are still gaps of knowledge as to the role of CB_1_R and the endocannabinoid system in the extensive pruning and development that is evident throughout adolescence.

## Addiction Vulnerability

A gateway drug hypothesis had long been proposed implying that adolescent cannabis use predisposes individuals to use other illicit drugs as adults, thereby increasing their vulnerability to substance use disorders (18) (Figure 2A). Although, the term “gateway” has sometimes been misinterpreted to imply that all individuals who use cannabis will directly abuse other drugs, this original hypothesis by Kandel (18) conducted on cohorts of high school students suggested that cannabis use is a critical illicit drug, intermediate in the transition from legal substance use (i.e., cigarettes and alcohol) to illicit drug use (i.e., heroin, amphetamines, and LSD). Over a quarter of individuals who progressed to illicit drug use had previous experience with marijuana while only 2–3% of legal drug users without marijuana experience progressed to illicit drug use. Subsequent longitudinal studies that tracked younger adolescents found that early cannabis use positively predicted cocaine and alcohol use across a 1-year period (19). Additional evidence that early-life cannabis consumption increases cocaine use later in life is supported by studies representing broad demographic populations (20), suggesting that these findings are likely generalizable.

Prospective longitudinal studies have also offered compelling evidence in support of the gateway drug hypothesis. A landmark 25 year-long study conducted on a birth cohort from New Zealand assessed associations between age of onset, and frequency of cannabis use, with the use and/or dependence of other substances (21). Even after controlling for a number of confounding variables, such as socio-economic background, other illicit substance use, family functioning, child abuse, and personality traits, early cannabis use was still significantly associated with subsequent drug abuse and dependence. Additionally this effect was age-related such that the association between cannabis use and the development of drug abuse and dependence declined with increasing age of initiation. An important strength of this study was that data collection extended beyond self-reports, and included parental interviews, medical records, psychometric assessment, and teacher reports. Twin-studies, which control for potential confounds such as genetics and shared environmental influences, have also confirmed that early adolescent onset of cannabis use increases the likelihood of developing drug dependence later in life (22).

**Figure 3:**
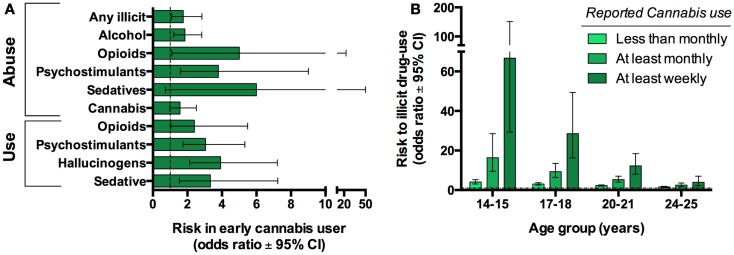
**Cannabis use is associated with progression to use other illicit substances in humans**. Twin-studies illustrate that cannabis users have an increased risk of developing substance abuse disorder compared to their discordant twin. Graph based on data adapted from Lynskey et al. (22) **(A)**. Cross-sectional studies reveal that earlier and more frequent cannabis use further increases this risk. Graph based on data adapted from Fergusson et al. (21) **(B)**.

One concern with human epidemiological studies is the inability to distinguish between casual and purely associative relationships. This is highlighted by a common-factor modeling study which suggests that correlations between cannabis and illicit drugs were principally attributed to other factors, namely an individual’s opportunity for and propensity to use drugs (23). Therefore, it has been argued that the transition from cannabis use to other drugs is not causal but is simply an expected sequence engaged by individuals that would normally go on to use other illicit drugs. Moreover, many teens who routinely smoke cannabis also use other drugs (e.g., alcohol and tobacco). While sequential transitions and the co-abuse of other drugs during such times could potentially contribute to enhance psychiatric risk, it is impossible to ignore the growing body of evidence that suggest a significant contribution of early adolescence cannabis specifically to the propensity to develop substance abuse disorders later in life even when controlling for other substances (21, 22) (Figure 3).

Animal studies allow the possibility to directly test the causal relationship between adolescent cannabinoid exposure and subsequent risk for drug addiction, independent of subject-specific factors that confound human investigations. Although a weakness of animal studies is that they do not mimic the complex nature of psychiatric disorder, specific phenotypes relevant to such disorders can be examined. In contrast to most psychiatric disorders, modeling addiction in animals is very predictive of the human condition through the use of self-administration paradigms wherein animals control their own drug intake. Under such conditions, adolescent exposure to THC reliably increases heroin self-administration (24, 25). In a similar investigation, performed in slightly older rats (approximately late adolescence), THC pre-exposure increased heroin self-administration when the contingency for heroin was fixed, but not when the work necessary to acquire heroin was progressively increased (26). Such findings imply that adolescent THC exposure increases the hedonic, but not motivational, aspects of heroin-seeking. Limited animal investigations have examined the sensitivity of early THC exposure to other “heavy” drugs of abuse such as cocaine, but the existing studies to date do highlight the generally enhancing effects of adolescent cannabinoid exposure on future drug-seeking behaviors, and experimentally support the gateway drug hypothesis.

Animal studies also provide specific insights about discrete neurobiological disturbances associated with developmental cannabinoid exposure. For example, adolescent THC increases inhibitory G-protein coupled signaling in the rodent midbrain, which by modulating dopaminergic projections, enhances mesolimbic dopamine, all adaptations strongly associated with enhanced reward (24). In addition, adolescent THC exposure increased mu opioid receptor function in the nucleus accumbens, a brain region central to reward and motivated behaviors, and these receptor impairments directly correlated to heroin intake (24). Moreover, increased gene expression of proenkephalin, an opioid neuropeptide that directly modulates heroin self-administration behavior, is also induced in the nucleus accumbens of adult rats with adolescent THC exposure (25). Enhanced cocaine self-administration has also been observed in female rats as a consequence of early-life exposure to the cannabinoid agonist CP-55,940 which was associated with altered striatal dopamine transporter binding in adulthood (27), and this transporter’s disturbance is highly implicated in addiction-related behaviors. Together these and other accumulating evidence in the literature emphasize that adolescent cannabinoids persistently change mesolimbic brain regions of the adult that sufficiently predict future self-administration behavior, a phenotype relevant to drug addiction vulnerability.

## Negative Affect and Anxiety

Another major question regarding the impact of adolescent cannabis relates to its role in negative affective disorders, such as major depressive disorder (MDD), which are increasingly burdensome worldwide. While equivocal, several longitudinal studies demonstrate an association between MDD and early-life exposure to cannabis. A large multi-cohort longitudinal investigation that examined the effects of adolescent cannabis use on depression and anxiety showed that frequent adolescent cannabis use increased depression and anxiety in early adulthood (28). Furthermore measures of depression and anxiety during adolescence did not predict cannabis use in young adults suggesting that this relationship was not simply due to premorbid differences. Similarly, while individuals who used cannabis during early teens did not differ in depression, suicidal ideation, or suicide attempts during adolescence, by early adulthood these individuals had significantly higher incidence of suicidal ideation and suicide attempts (29). A consistent observation was reported in another large longitudinal investigation, which found that adults with early cannabis use had increased suicidal behaviors (30). Altogether these findings emphasize the important contribution of early cannabis exposure to MDD and suicidal ideation. Importantly, accumulating evidence also implies that both adolescent exposure and the continued use during adulthood are required for these associations (31, 32) suggesting that disease may be mitigated with cannabis cessation.

It is important to note that although most studies to date imply an association of early cannabis with negative affective disorders, the longitudinal cohort investigation by Harder et al. (33) did not find any difference in depression or anxiety either during early adolescence or at the last follow-up in adulthood. This inconsistency may be due to the study’s lenient definition of a “cannabis user,” which included any participant who ever smoked cannabis prior to age 17 ( ∼50% population). Although additional studies are needed to understand the long-term causative effects of adolescent cannabis on negative affect, a preponderance of the evidence accrued thus far strongly suggests a correlation between these two factors.

Future longitudinal studies are clearly still needed to examine the contribution of the developmental period of onset and cessation of cannabis to the risk of negative affect. In addition, *in vivo* neuroimaging in humans can also offer much needed neurobiological insights. Evidence already exists demonstrating volumetric impairments in the amygdala, a brain region central to affective and addictive disorders, in cannabis users during early (34), and late (35) adolescence. Similarly, structural changes in the hippocampus, which is linked to depression (36), has been reported in individuals with cannabis use during late adolescence (35, 37).

The use of animal models has also helped to fill gaps of knowledge regarding the direct link between early-life cannabis use and negative affect and anxiety. Such experimental studies have demonstrated that early exposure to cannabinoids directly leads to dysregulation of emotional processes and induces depressive-like phenotypes later in life. For instance, escalating doses of THC to adolescent rats decreases sucrose preference, a measure of anhedonia (38). Other behavioral strategies such as the forced-swim test used to measure depression-related symptoms also reveal a pro-depressive phenotype directly associated with adolescent THC (39), although these effects generally appear stronger in females (38, 40). These findings suggest that adolescent cannabinoid exposure could affect the liability to mood disorders later in life, and the potential gender differences may relate in those well-documented in human depression.

Altered anxiety-like behavior as a consequence of adolescent cannabinoid exposure is also apparent in experimental animals though the relationship is not straightforward *per se*. Anxiogenesis or anxiolysis has been reported depending on the period of cannabinoid exposure and the specific task used to model anxiety. For example, chronic exposure to cannabinoid agonists – such as THC, CP-55,940, or WIN-55,212-2 – during mid- to late-adolescence, increases social anxiety as measured with a social recognition task (41–44). Other measurements of stress that do not rely on social interaction, such as the open-field and elevated plus-maze tests, indicate varying degrees of anxiolysis, not anxiogenesis (41, 45, 46). These anxiolytic effects were observed after mid- to late-adolescent exposure, whereas earlier, pre-pubertal exposures (PND 15–40) were anxiogenic (47). Consistent with the notion of critical periods, persistent alterations in anxiety almost exclusively occur after early-life exposure and not in animals exposed as adults (39).

Few animal experimental studies have specifically focused on examining neurobiological mechanisms associated with regulation of emotion in association with adolescent cannabinoid exposure. Of the studies, Page et al. (48) demonstrated that administration of the cannabinoid agonist WIN-55,212-2 to adolescents, as compared to adult rats, more profoundly and persistently disrupted cells in the locus coeruleus, a midbrain region that contains noradrenergic neurons and is implicated with depression and anxiety. Similarly, adolescent animals treated with WIN-55,212-2 exhibit altered midbrain neuronal firing characteristics that were not observed in adult-exposed rats (39). Specifically, the cannabinoid treatment resulted in hyperactivity of the noradrenergic neurons concomitant with hypoactivity of serotonergic cells (39). Such neuroadaptations would be predictive of enhanced anxiety and depression-like behavior as a consequence of early cannabinoid exposure.

## Schizophrenia and Schizoaffective Disorders

Although a small fraction of teens that use cannabis develop schizoaffective disorders, a number of epidemiological studies repeatedly demonstrate elevated risk to develop these psychiatric disorders in association with early-life cannabis use. Longitudinal studies assessing the relationship between early-life cannabis exposure and schizotypal personality disorder demonstrated that early adolescent use increases adulthood symptomatology (49). Moreover, the presence and severity of schizophrenic endophenotypes, such as psychotic symptoms and prepulse inhibition, were predicted by adolescent cannabis use (50, 51).

The first longitudinal studies demonstrating an association between cannabis use before adulthood and schizophrenia were conducted in Swedish conscripts (52, 53) Although no information was known about the individuals before conscription, subjects reporting previous cannabis use at the time of conscription were significantly more likely to be diagnosed with schizophrenia later in life. These findings were replicated in multiple studies emphasizing the reproducible relationship between adolescent cannabis use and increased schizophrenia symptoms in adulthood (54, 55).

Although it is challenging to model schizophrenia in animals, phenotypes related to this disorder may be studied. Animals exposed to cannabinoids during adolescence demonstrate increased schizoaffective-like phenotypes, such as impaired sensorimotor gating, which, similar to humans, results in decreased prepulse inhibition (45). Consistent with the notion that developmental cannabinoids induce a schizophrenia-like phenotype, acute administration of the anti-psychotic haloperidol normalized prepulse inhibition in the cannabinoid-exposed rats (47).

Since not all cannabis users develop schizophrenia, early cannabis use likely interacts with other factors to facilitate the emergence of this disease (56). Accumulating data in recent years highlight that the association between early cannabis exposure and vulnerability to schizophrenia is related to individual genetics. Pioneering studies by Caspi et al. (57) demonstrated that the relationship between adolescent cannabis use and schizophreniform disorder, as well as the presence of various psychotic symptoms, was attributable to the presence of a functional polymorphism in the catechol-O-methyltransferase (*COMT*) gene. This enzyme degrades catecholamines, such as dopamine, and this functional variant (COMTvaline^158^) catabolizes this neurotransmitter more rapidly than the methionine allele (58). In cannabis users, schizophreniform disorder is predominantly observed in persons with at least one copy of the polymorphic *COMT* gene (59–61). Moreover, clinical laboratory experiments show that THC’s acute psychotomimetic effects are moderated by this *COMT* SNP with THC-induced psychotic-like experiences and cognitive impairments being more pronounced in individuals with the valine^158^ allele (62). Animal models also confirm a link between the genetic disturbance of *COMT* and developmental cannabis such that adolescent THC exposure in transgenic mice lacking endogenous *COMT* synergistically impacts behaviors relevant to schizophrenia (63). Overall, these human and animals studies highlight the significant association between early cannabis exposure and schizophrenia, supporting the so-called two-hit hypothesis which posits that both genetics and early environmental factors enhance individual risk to psychiatric illnesses.

## Phytocannabinoids and Psychiatric Vulnerability

It is important to emphasize that while most studies focused on THC to understand the long-term impact of cannabis, the plant produces at least 70 cannabinoids (64). To date the most studied phytocannabinoid aside from THC is cannabidiol (CBD), the second major constituent of the cannabis plant. Interestingly, in contrast to THC, CBD appears to have more protective effects relevant to addiction, cognition, and negative affect. For example, CBD inhibits drug-seeking behavior associated with heroin-relapse in rats (65), reduces cigarette intake (66), and inhibits morphine reward (67). It also has anti-psychotic properties (68, 69) and reduces anxiety behavior in rodents (70) and humans (66). Most of these investigations, however, were carried out in adults. No published study to date has examined CBD in relation to adolescent development and subsequent behavioral consequences in later life. As such, it remains to be explored whether the potential positive effects of CBD on brain function seen in adults would also be evident with adolescent exposure. One intriguing consideration about CBD relevant to the developing brain is that cannabis plants today ingested by teens are grown for high THC, but low CBD content (71). This significant change in the THC:CBD ratio could reduce a normally apparent protective constituent of cannabis. The fact that so little is known about CBD and the developing brain highlights the need for research about this and other phytocannabinoids to more fully understand the impact of cannabis to psychiatric vulnerability.

## Conclusion

The high prevalence of cannabis use among teens and the increasing number of states in the USA that legalize cannabis for both medicinal and recreational purposes are concerning given the surprisingly limited information known about the impact of cannabis on the developing brain and individual susceptibility. Though a causative relationship cannot be determined between marijuana’s glamorization and its increasing use in teenagers, important lessons can be learned from the major inroads made in reducing cigarette use in youths such as interventions through campaigns that made smoking less socially accepted. Based on the current evidence available from human and animal models, it is evident that cannabis use during adolescent development increases risk of psychiatric diseases such as drug addiction and schizoaffective disorders with genetic interactions. No convincing data exist to support one “common cause” that exclusively predicts which individuals using cannabis as teens will progress to addiction and psychiatric disorders later in life versus those who do not. Psychiatric diseases, such as those discussed in this review, are complex and multifactorial. Indeed, the complex transition from early cannabis use to subsequent psychiatric illness involves multiple factors such as genetics, environment, time period of initiation and duration of cannabis use, underlying psychiatric pathology that preceded drug use, and combined use of other psychoactive drugs. Whether the early onset of cannabis use relates to preexisting pathology that is then exacerbated by the drug is still debated. Additionally, it remains uncertain whether there exist specific critical windows of vulnerability during different phases of adolescent development relevant to the long-term trajectory of risk in adulthood. Longitudinal investigations, making use of neuroimaging and genetics, alongside concurrent studies in animal models are needed to fully elucidate molecular mechanisms that could provide novel treatment interventions for individuals with psychiatric disease and comorbid adolescent cannabis use.

## Conflict of Interest Statement

The authors declare that the research was conducted in the absence of any commercial or financial relationships that could be construed as a potential conflict of interest.
